# Emotion recognition profiles in clusters of youth based on levels of callous-unemotional traits and reactive and proactive aggression

**DOI:** 10.1007/s00787-022-02079-3

**Published:** 2022-09-20

**Authors:** Renee Kleine Deters, Jilly Naaijen, Nathalie E. Holz, Tobias Banaschewski, Ulrike M. E. Schulze, Arjun Sethi, Michael C. Craig, Ilyas Sagar-Ouriaghli, Paramala Santosh, Mireia Rosa, Josefina Castro-Fornieles, María José Penzol, Celso Arango, Daniel Brandeis, Barbara Franke, Jeffrey C. Glennon, Jan K. Buitelaar, Pieter J. Hoekstra, Andrea Dietrich

**Affiliations:** 1 Department of Child and Adolescent Psychiatry, University Medical Center Groningen, University of Groningen, Groningen, The Netherlands; 2 Accare Child Study Center, Groningen, The Netherlands; 3 Department of Cognitive Neuroscience, Donders Institute for Brain, Cognition and Behaviour, Radboud University Medical Center, Nijmegen, The Netherlands; 4 Department of Child and Adolescent Psychiatry and Psychotherapy, Medical Faculty Mannheim, Central Institute of Mental Health, Heidelberg University, Mannheim, Germany; 5 Department of Child and Adolescent Psychiatry and Psychotherapy, University Hospital, University of Ulm, Ulm, Germany; 6 Department of Forensic & Neurodevelopmental Sciences, Institute of Psychiatry, King’s College London, Psychology & Neuroscience, London, UK; 7 Department of Child & Adolescent Psychiatry, Institute of Psychiatry, King’s College London, Psychology & Neuroscience, London, UK; 8 Centre for Interventional Paediatric Psychopharmacology and Rare Diseases (CIPPRD), National and Specialist Child and Adolescent Mental Health Services, Maudsley Hospital, London, UK; 9 Department of Child and Adolescent Psychiatry and Psychology, Clínic Institute of Neurosciences, Hospital Clínic de Barcelona, IDIBAPS, Barcelona, Spain; 10 Department of Child and Adolescent Psychiatry and Psychology, Clínic Institute of Neurosciences, Hospital Clínic de Barcelona, CIBERSAM, IDIBAPS, University of Barcelona, 2017SGR881 Barcelona, Spain; 11 Child and Adolescent Psychiatry Department, Institute of Psychiatry and Mental Health, Hospital General Universitario Gregorio Marañón School of Medicine, IiSGM, CIBERSAM, Universidad Complutense, Madrid, Spain; 12 Department of Child and Adolescent Psychiatry and Psychotherapy, Psychiatric Hospital, University of Zurich, Zurich, Switzerland; 13 Department of Human Genetics, Donders Institute for Brain, Cognition and Behaviour, Radboud University Medical Center, Nijmegen, The Netherlands; 14 Department of Psychiatry, Donders Institute for Brain, Cognition and Behaviour, Radboud University Medical Center, Nijmegen, The Netherlands; 15 Conway Institute for Biomolecular and Biomedical Research, University College Dublin, Belfield, Dublin Ireland; 16 Karakter Child and Adolescent Psychiatry University Center, Nijmegen, The Netherlands

**Keywords:** Callous-unemotional traits, Reactive aggression, Proactive aggression, Emotion recognition, Disruptive behavior problems

## Abstract

Youth with disruptive behavior showing high callous-unemotional (CU) traits and proactive aggression are often assumed to exhibit distinct impairments in emotion recognition from those showing mainly reactive aggression. Yet, reactive and proactive aggression and CU traits may co-occur to varying degrees across individuals. We aimed to investigate emotion recognition in more homogeneous clusters based on these three dimensions. In a sample of 243 youth (149 with disruptive behavior problems and 94 controls) aged 8–18 years, we used model-based clustering on self-report measures of CU traits and reactive and proactive aggression and compared the resulting clusters on emotion recognition (accuracy and response bias) and working memory. In addition to a Low and Low-Moderate symptom cluster, we identified two high CU clusters. The CU-Reactive cluster showed high reactive and low-to-medium proactive aggression; the CU-Mixed cluster showed high reactive and proactive aggression. Both CU clusters showed impaired fear recognition and working memory, whereas the CU-Reactive cluster also showed impaired recognition of disgust and sadness, partly explained by poor working memory, as well as a response bias for anger and happiness. Our results confirm the importance of CU traits as a core dimension along which youth with disruptive behavior may be characterized, yet challenge the view that high CU traits are closely linked to high proactive aggression per se. Notably, distinct neurocognitive processes may play a role in youth with high CU traits and reactive aggression with lower versus higher proactive aggression.

**Supplementary Information:**

The online version contains supplementary material available at 10.1007/s00787-022-02079-3.

## Introduction

Antisocial and disruptive behaviors are among the most frequent reasons for referral to child and adolescent mental health services [1]. These behaviors have been consistently linked to difficulties in emotion recognition ability, which is fundamental to behavioral regulation in social contexts [2]. Yet, antisocial and disruptive behaviors are highly heterogeneous and encompass multiple dissociable dimensions reflecting distinct social and behavioral issues. A key dimension is callous-unemotional (CU) traits, referring to specific affective (lack of guilt, shallow emotions) and interpersonal (lack of empathy, callousness) traits, which predispose to severe and persistent antisocial behavior and aggression [3]. Aggression, in turn, may be characterized as reactive, reflecting responses to perceived threat or provocation, or proactive, reflecting goal-directed and planned aggression closely associated with the presence of CU traits [4, 5].

Accordingly, most studies particularly distinguish between reactive aggression versus CU traits and proactive aggression with respect to emotion recognition abilities [6, 7]. Yet, some recent studies point to distinct neural correlates for CU traits versus proactive aggression [8–10]. In addition, a person-oriented approach on clinic-referred youth with disruptive behavior resulted in different clusters with varying levels of CU traits and aggression, one of which exhibited high CU traits and reactive aggression yet low proactive aggression [11]. As dimensional approaches do not consider that both aggression dimensions and CU traits may co-occur in varying degrees across individuals, the characterization of more homogeneous subgroups regarding these dimensions may increase our understanding of underlying neurocognitive mechanisms.

Still, so far, most studies related emotion recognition deficits to univariate, variable-based measures of CU traits and reactive and proactive aggression, with only few studies including all three dimensions. Specifically, a failure to recognize facial expressions of distress (i.e. fear) is a well-established finding in youth with high levels of CU traits [12], with evidence for a partly shared genetic etiology [13]. In contrast, disruptive behavior with low CU traits has been linked to a bias to interpret ambiguous or neutral facial expressions as hostile (i.e. as anger), indicating elevated threat sensitivity and increased reactive aggression [7].

Few studies have focused on emotion recognition regarding reactive versus proactive aggression, with inconclusive results. In one study, offenders showing proactive violence performed similar to controls, whereas offenders showing reactive violence performed worse in recognizing anxiety, disgust, and sadness, and had a tendency to interpret non-anger emotions as anger [14]. In addition, better (overall) emotion recognition has also been related to more proactive, but not reactive aggression [15], while proactive aggression has also been associated with impaired emotion recognition in youth with conduct disorder [16].

Impaired emotion recognition may in part be explained by working memory deficits [17, 18]. Specifically, a high working memory load during a dual task has been shown to interfere with the correct categorization of facial expressions, which may be partly related to task demands, but may also be intrinsic to the decoding of social cues [19]. Notably, while poorer working memory has been associated with increased reactive and proactive aggression, better working memory has also been associated with increased proactive aggression [20, 21]. Findings regarding CU traits are limited and do not support a link with working memory ability [22, 23]. Thus, the association between working memory and emotion recognition abilities in the context of CU traits and aggression remains unclear.

In the current study, we aimed to identify multiple homogeneous subgroups with respect to the aforementioned traits using model-based clustering, a person-based data-driven approach accounting for heterogeneity in the co-occurrence of multiple traits within individuals. We included both clinic-referred children and adolescents with disruptive behavior problems and control youths to account for the fact that these behavioral traits exist on a continuum across referred and non-referred samples [24]. We then compared working memory and emotion recognition (accuracy and response bias), the latter with and without adjusting for working memory, across the resulting clusters, as well as misclassification of emotions. Overall, we expected the largest emotion recognition deficits particularly in youth with high CU traits and/or proactive aggression.

## Methods and materials

### Participants

Participants were recruited across nine sites in Europe as part of the joint European Matrics and Aggressotype projects (http://www.matrics-project.eu; http://www.aggressotype.eu; also [25]). Out of 283 participants, we selected those who completed all three clustering measures, resulting in a sample of 243 participants aged 8–18 years, with 94 controls (*n*_male_ = 53, *M*_age_ = 3.4, *SD* = 2.5) and 149 cases (*n*_male_ = 125, *M*_*age*_ = 12.9, *SD* = 2.8). Cases were required to have a current diagnosis of ODD and/or CD based on the Kiddie Schedule for Affective Disorders and Schizophrenia (K-SADS) [26], and/or clinical levels of disruptive behavior defined as clinical scores (T ≥ 70) on the parent-rated aggressive behavior and/or rule-breaking behavior subscales of the Child Behavior Checklist (CBCL) [27]. The K-SADS was also used to assess the presence of ADHD symptom counts and diagnoses. Controls were recruited from schools, cases from centers for child and adolescent psychiatry. Exclusion criteria were IQ < 80, or any DSM-5 [28] diagnosis in controls, screened for by using the K-SADS. Participants using psychotropic medication were required to have used a stable dose during at least 2 weeks prior to participation; stimulants were abstained at one of the test-sites (Nijmegen) during the test day. Written informed consent and/or assent was obtained from participants and their parents or legal guardian, in accordance with national regulations. Ethical approval was obtained at all sites from local ethics committees.

## Measures

### CU traits

CU traits were assessed by the self-reported Inventory of Callous-Unemotional Traits (ICU) [29], with 24 items rated on a four-point scale from 0 (not at all true) to 3 (definitely true). The total score represents the sum of all item values (in the current sample range 9–56; Cronbach’s *α* = 0.79).

### Aggression

Reactive and proactive aggression were assessed by the self-reported Reactive Proactive Aggression Questionnaire (RPQ) [4]. Items were rated on a three-point scale according to frequency, from 0 (never) to 2 (often), with 12 items for proactive aggression (range 0–22, Cronbach’s α = 0.88) and 11 items for reactive aggression (range 0–21, Cronbach’s *α* = 0.88).

### Working memory and emotion recognition

**Fig. 1:**
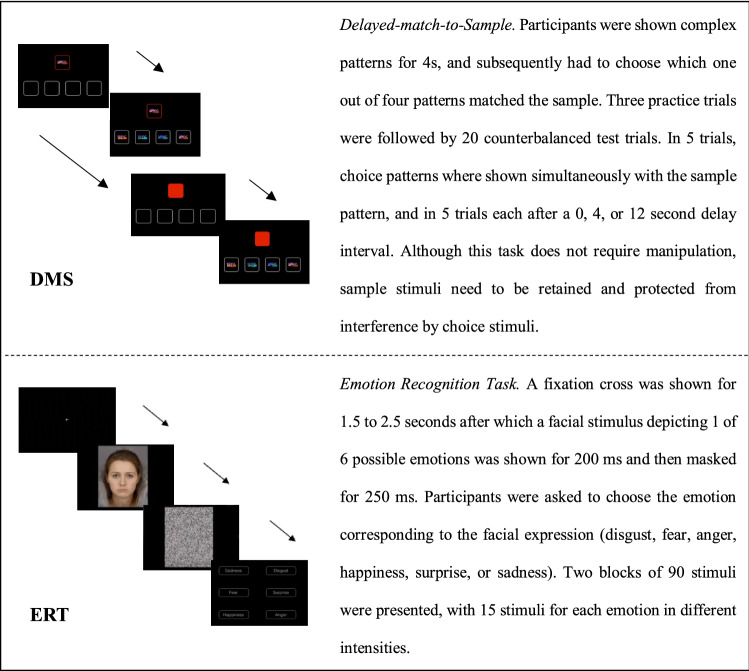
Description of the Cambridge Neuropsychological Test Automated Battery (CANTAB) tasks used in the current study

Visual working memory was assessed with the Delayed-Match-to-Sample task, emotion recognition with the Emotion Recognition Task (see Fig. 1 for detailed descriptions). Both are part of the Cambridge Neuropsychological Test Automated Battery (CANTAB) [30], a computerized program with internal consistency coefficients ranging from 0.73 to 0.95 and good validity [31]. Response accuracy (percentage of correct answers) was used as the main outcome measure for both tasks; for the Emotion Recognition Task, response accuracy was calculated for each target emotion.

## Statistical analysis

All analyses were performed with the R statistical program (version 3.6.1) [32].

### Model-based clustering

We performed model-based clustering (MBC) on standardized measures of CU traits, reactive, and proactive aggression using the mclust package in R [33]. MBC models the number and type of multivariate unobserved subpopulations (i.e. clusters) underlying the observed data. MBC addresses the uncertainties inherent to common clustering simultaneously testing the relative fit of 10 models that vary in their assumptions about the structure of the data, with the numbers of clusters allowed to vary from 1 to 9. The Bayesian Information Criterion (BIC) [34] is used to evaluate model fit and to subsequently choose the best model. We calculated the average posterior probabilities for each cluster as an indication of classification certainty, with a value > 70% suggested as indicating clear classification [35].

### Cluster characteristics

Cluster differences regarding phenotypic and clinical information were analyzed with one-way analyses of variance (ANOVAs) for continuous variables, followed by pairwise comparisons to determine specific differences between clusters, and with Pearson’s Chi-square tests for categorical variables.

### Working memory and emotion recognition

The main outcome measures (response accuracy) of the working memory and emotion recognition tasks were screened for outliers, which were defined as a *z* score ≥|3.0| on the working memory task and two or more *z* scores ≥|3.0| on the emotion recognition task (as emotions were displayed in random order; interpreted as indicative of insufficient task effort). First, linear regression analysis was used for the analysis of working memory between clusters. Then, emotion recognition was compared between clusters using a linear mixed-effects model (LME4 package) [36] to account for within-subject variance (emotions nested within subjects). Next, we repeated the mixed-effects model with working memory now included as a covariate of interest. Post-hoc comparisons between all clusters were performed, using the false discovery rate (FDR) *q* values to account for multiple comparisons. Finally, sensitivity analyses were performed by, respectively, adding medication status (yes/no), site, and ADHD symptom counts as covariates of non-interest. Age, sex, and IQ were added as covariates of non-interest in all main analyses. Effect sizes are reported as ‘*r*’ (small ≥ 0.10, medium ≥ 0.30, large ≥ 0.50) [37].

### Response bias

To investigate differences in response patterns, we calculated the overall frequency of commission errors for each emotion (i.e. how often that emotion was incorrectly chosen) as a percentage of all non-target trials. We compared these percentages between clusters using linear regression analyses with the percentage of commission errors for each emotion separately as outcome measure, using the FDR *q* value to account for multiple comparisons.

## Results

### Model-based clustering

The best-fitting model had a BIC of –1653.036 and included four clusters ellipsoidal in distribution, with variable volume and orientation [33]. The second-best model was a three-cluster solution with a BIC value of –1693.346. A difference in BIC of 4 is considered as positive evidence in favor of the model with the greater BIC, this pointing to a better fit of the four-cluster model. The average classification certainty was 88%.

**Fig. 2:**
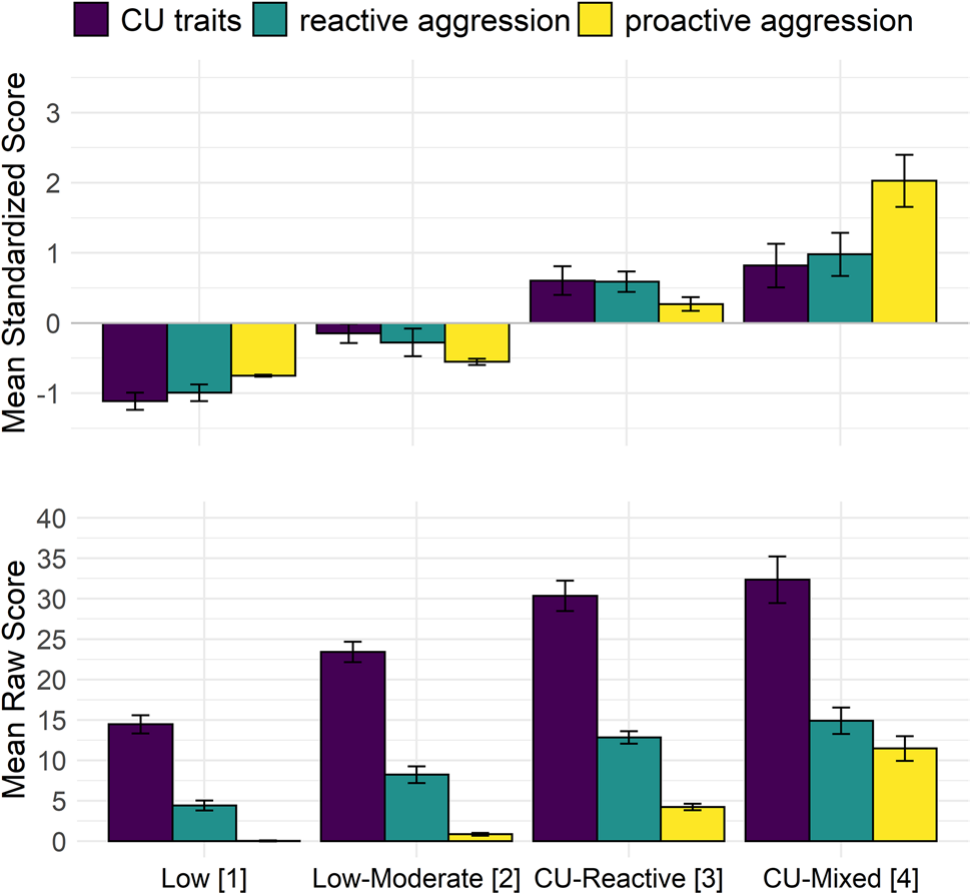
Standardized and raw means of variables used for clustering. *CU* callous-unemotional. Error bars indicate 95% confidence intervals

See Fig. 2 for a graphical representation of the clusters (both raw and standardized scores for presentation purposes). The ‘Low’ [1] cluster (classification certainty 89%) exhibited low levels of CU traits and reactive and proactive aggression; the ‘Low-Moderate’ [2] cluster (classification certainty 82%) exhibited low-medium levels of CU traits and reactive aggression and low proactive aggression; the ‘CU-Reactive’ [3] cluster (classification certainty 90%) exhibited high levels of CU traits, (moderately) high reactive aggression, and low-to-medium proactive aggression; The ‘CU-Mixed’ [4] cluster (classification certainty 95%) exhibited high levels of CU traits, high reactive aggression, and high proactive aggression.

**Fig. 3:**
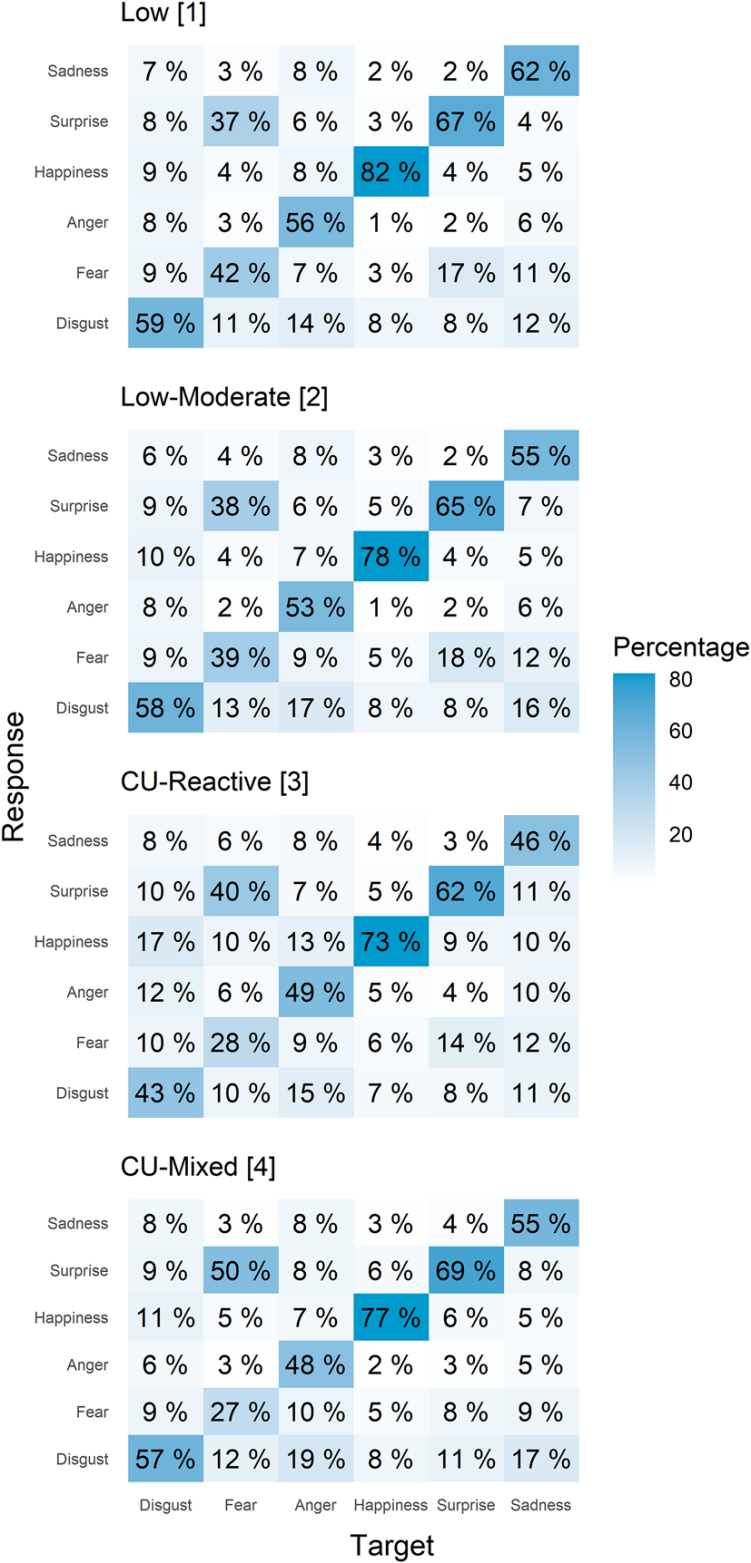
Confusion matrices showing emotion (mis)identification patterns. Values on the diagonal indicate the percentage of correct responses

### Cluster characteristics

CANTAB data were missing for a subset of participants (*n* = 51) included in the cluster analysis See Table 1 for cluster characteristics and differences for the sample who completed the CANTAB. The clusters differed in level of CU traits [*F*(3, 188) = 59.5, *p* < 0.001], reactive [*F*(3, 188) = 53.7, *p* < 0.001], and proactive aggression [*F*(3, 188) = 220.1, *p* < 0.001]. While mean levels of CU traits and reactive aggression did not differ between the CU-Reactive and CU-Mixed clusters, the CU-Mixed cluster showed a higher level of proactive aggression. The proportion of cases differed between clusters [*χ*^2^ = 63.4, df = 3, *p* < 0.001], being highest in the CU-Reactive and CU-Mixed clusters. The clusters included different proportions of males [*χ*^2^ = 14.3, df = 3, *p* = 0.003], with the highest proportion in the CU-Reactive cluster. The number of participants with ODD [*χ*^2^ = 34.3, df = 3, *p* < 0.001] and CD [*χ*^2^ = 36.2, df = 3, *p* < 0.001] differed across clusters, with the lowest proportions in the Low cluster. The number of participants with ADHD [*χ*^2^ = 20.3, df = 3, *p* < 0.001] and mean ADHD symptom count [*F(*3, 188) = 16.5, *p* < 0.001] also differed between the clusters, with the highest numbers in the CU-Reactive cluster. The proportion of individuals using psychotropic medication was different across clusters [*χ*^2^ = 35.6, df = 3, *p* < 0.001], with the highest proportion in the CU-Reactive and CU-Mixed clusters. Mean age [*F(*3, 188) = 4.0, *p* = 0.008] and IQ [*F(*3, 187) = 5.8, *p* < 0.001] differed between clusters, with the highest age (although not significantly older than the Low cluster) and the lowest IQ (while still in the normal range) in the CU-Mixed cluster. Supplementary table 1 contains the cluster characteristics based on the whole sample.

**Table 1:** Cluster characteristics

Characteristic	Low [1] *n* = 45	Low-Moderate [2] *n* = 64	CU-Reactive [3] *n* = 61	CU-Mixed [4] *n* = 22	Pairwise comparisons
*n* cases (%)^a^	9 (20%)	30 (47%)	54 (89%)	20 (91%)	3,4 > 2 > 1
*n* male (%)^a^	24 (53%)	41 (64%)	52 (85%)	17(77%)	3 > 1,2
*n* ODD (%)^a^	5 (11%)	18 (28%)	38 (62%)	12 (54%)	3,4 > 1,2
*n* CD (%)^a^	2(4%)	7 (11%)	22 (36%)	13 (59%)	3,4 > 1,2
*n* ADHD (%)^a^	1 (2%)	7 (11%)	20 (33%)	3 (14%)	3 > 1,2
*n* medication (%)^a^	3 (7%)	12 (19%)	32 (53%)	12(54%)	3,4 > 1,2
Age, M (SD)^b^	13.2 (2.5)	12.6 (2.5)	12.8 (2.8)	14.7 (2.4)	4 > 1,2,3
IQ, M (SD)^b^	109 (12)	104 (11)	101 (11)	98 10)	1 > 2 > 4; 1 > 3
Aggression, M (SD)^b^	55 (9)	63 (13)	74 (12)	74 (12)	3,4 > 2 > 1
Rule-breaking, M (SD)^b^	54 (6)	59 (10)	67 (8)	71 (11)	3,4 > 2 > 1
ADHD symptoms, M (SD)^b^	0.9 (2.8)	2.6 (4.4)	6.8 (5.6)	3.8 (4.8)	3 > 1,2,4; 4 > 1
Clustering measures					
CU traits, M (SD)^b^	14.2 (4.3)	23.1 (5.8)	30.1 (8.5)	21.8 (7.6)	3,4 > 2 > 1
Reactive aggression, M (SD)^b^	4.6 (2.2)	8.1 (4.4)	12.7 (3.5)	13.8 (4.6)	3,4 > 2 > 1
Proactive aggression, M (SD)^b^	0.0 (0.2)	0.9 (0.8)	4.1 (1.7)	10.1 (3.8)	4 > 3 > 2 > 1

*ODD* oppositional defiant disorder; *CD* conduct disorder; *CU* callous-unemotional; *ADHD* attention deficit/hyperactivity disorder. Aggression and Rule-breaking as assessed by respective subscales of the child behavior checklist (*CBCL*) [27]; CU traits as assessed by the Inventory of callous-unemotional traits (ICU) [29] Reactive and Proactive Aggression as assessed by the reactive-proactive aggression questionnaire (RPQ) [4]. The clusters are numbered to indicate significant pairwise comparisons

^a^Cluster differences assessed by Pearson’s Chi-square test

^b^Cluster differences assessed by one-way analyses of variance (ANOVA)

All pairwise comparisons significant at *p* < 0.05

### Working memory

We excluded 2 participants as outliers on the working memory task. See Table 2 for mean cluster performance and differences. The CU-Mixed cluster showed lower response accuracy compared to the Low and Low-Moderate clusters [*b* = – 10.0, *t*(182) = – 2.8, *q* = 0.015, *r* = 0.20; *b* = – 9.9, *t*(182) = – 3.0, *q* = 0.015, *r* = 0.22, respectively], while the CU-Reactive cluster only showed lower response accuracy compared to the Low-Moderate cluster [*b* = – 5.6, *t*(182) = – 2.4, *q* = 0.040, *r* = 0.18].

**Table 2:** Working memory and emotion recognition performance

Measure	Low [1]	Low-Moderate [2]	CU-Reactive [3]	CU-Mixed [4]	Pairwise comparisons
DMS, M (SD)	85 (12)	84 (12)	78 (15)	77 (15)	4 < 1^a^, 2^a^; 3 < 1^ab^
ERT					
Disgust, M (SD)	59 (19)	58 (22)	43 (23)	57 (26)	3 < 1^c^,2
Anger, M (SD)	56 (11)	53 (11)	49 (17)	48 (16)	–
Fear, M (SD)	42 (10)	39 (21)	28 (17)	27 (20)	3,4 < 1,2
Happiness, M (SD)	81 (12)	78 (12)	73 (10)	77 (15)	–
Surprise, M (SD)	67 (16)	65 (19)	62 (22)	69 (20)	–
Sadness, M (SD)	62 (16)	55 (17)	45 (20)	55 (17)	3 < 1^c^, 2^c^

All scores reflect % correct. *DMS* delayed-match-to-sample; *ERT* Emotion Recognition Task

^a^No longer significant when medication was added to the model

^b^No longer significant when ADHD symptom count was added to the model

^c^No longer significant when DMS (working memory) score was assed to the model

All pairwise differences significant at *q* < 0.05

Sensitivity analyses indicated a main effect of medication use (yes/no) [*b* = – 5.2, *t*(178) = – 2.3, *p* = 0.021, *r* = 0.17] and ADHD symptom count [*b* = – 0.432, *t*(181) = 2.0, *p* = 0.038, *r* = 0.15], but not of site, with worse performance in participants using medication and with more ADHD symptoms (note that the Low cluster only contained four participants using medication). All group differences regarding working memory performance became insignificant when medication was added to the model. When adding ADHD symptoms counts, only the difference between the Low-Moderate and CU-Reactive cluster became insignificant.

### Emotion recognition

There were no outliers on the emotion recognition task. See Table 2 for mean performance and cluster differences. The CU-Reactive and CU-Mixed cluster showed lower response accuracy regarding fear compared to the Low [*b* = –12.6, *t*(837) = – 3.6 *q* = 0.001, *r* = 0.12; *b* = – 16.1, *t*(855) = – 3.5, *q* = 0.001, *r* = 0.12], and Low-Moderate [*b* = – 11.1 *t*(850) = – 3.6, *q* = 0.001, *r* = 0.12; *b* = – 14.6, *t*(863) = – 3.4, *q* = 0.01, *r* = 0.11] clusters. In addition, the CU-Reactive cluster was worse in recognizing disgust and sadness compared to the Low [*b* = –11.2, *t*(837) = – 3.2, *q* = 0.004, *r* = 0.11; *b* = – 11.7 *t*(837) = – 3.4, *q* = 0.005, *r* = 0.12] and Moderate [*b* = – 14.4 *t*(850) = – 4.7, *q* < 0.001, *r* = 0.15; *b* = – 11.2, *t*(850) = – 2.8, *q* = 0.004, *r* = 0.10] clusters.

When working memory was added to the model as a covariate, results indicated an association with overall emotion recognition across clusters [*b* = 0.36, *t*(180) = 7.0, *p* < 0.001, *r* = 0.46]. Differences between the CU-Reactive and Low and Low-Moderate clusters regarding sadness recognition became insignificant when working memory was added; for disgust, only the difference with the Low cluster became insignificant.

Sensitivity analyses indicated that neither medication use, ADHD symptom counts, nor site were related to emotion recognition. All cluster differences remained significant when these variables were added to the model.

### Response bias

The frequency of commission errors for anger and happiness was higher in the CU-Reactive cluster compared to the Low [*b* = 0.03, *t*(188) = 3.8, *q* < 0.001, *r* = 0.27; *b* = 0.05, *t*(188) = 3.9, *q* < 0.001, *r* = 0.27], Low-Moderate [*b* = 0.04, *t*(188) = 4.6, *p* < 0.001, *r* = 0.32; *b* = 0.05, *t*(188) = 4.3, *q* < 0.001, *r* = 0.30], and CU-Mixed [*b* = 0.04, *t*(188) = 3.3, *p* = 0.002, *r* = 0.24 *b* = 0.05, *t*(188) = 2.6, *q* = 0.019, *r* = 0.19] clusters. In addition, the CU-Reactive and CU-Mixed clusters more often responded with surprise compared to the Low-Cluster [*b* = 0.03, *t*(188) = 2.3, *q* < 0.041, *r* = 0.17; *b* = 0.05, *t*(188) = 2.9, *q* = 0.024, *r* = 0.21]; the CU-Mixed cluster also compared to the Low-Moderate cluster [*b* = 0.03, *t*(188) = 2.3, *q* < 0.041, *r* = 0.17] ( Fig. 3).

## Discussion

We examined emotion recognition and working memory in four subgroups of youth with distinct levels of CU-traits and reactive and proactive aggression, derived from model-based clustering. While the CU-Reactive and CU-Mixed clusters both exhibited high levels of CU traits and reactive aggression and showed impaired fear recognition relative to Low and Low-Moderate clusters, only the CU-Mixed cluster showed markedly high proactive aggression. Notably, the CU-Reactive cluster also showed poor recognition of sadness and disgust, as well as an increased tendency to respond with anger (possibly reflecting increased threat sensitivity) and happiness. Thus, our results confirm impaired fear recognition as a main characteristic related to high CU traits [6]. Yet, our study also points to a distinction between two subgroups of youth with high CU traits and reactive aggression; those with relatively low proactive aggression and a wider range of difficulties and biases in emotion recognition likely associated with increased threat sensitivity, who seem to reflect a more emotionally impulsive subgroup, versus those with high proactive aggression, likely associated with fearlessness and reduced threat sensitivity, who seem to reflect a more instrumental aggressive subgroup [24]. Overall, our findings indicate that low versus high proactive aggression in youth with high CU traits and reactive aggression may account for distinct emotion recognition patterns.

Our results are in line with previously identified clusters of youth showing high CU traits but distinct levels of proactive aggression [11], as well as with evidence for similar levels of CU traits in mainly reactive versus mixed reactive/proactive aggression clusters [14, 38]. Yet, our findings challenge the assumption that youth with disruptive behavior and high CU traits generally also exhibit high proactive aggression [3, 6]. Still, our results may be seen in light of differences in sample characteristics. Many studies have used median-split or arbitrary cut-off scores for the ICU (including cut-off scores of 28, 30, 38, and 44) [5, 39]. According to some of these cut-off scores, the current study would have included only few individuals with high CU traits, thus suggesting other studies may have included more severely affected populations. Perhaps, individuals with the highest levels of CU traits may be primarily those who also exhibit proactive aggression [40].

Our finding of impaired fear recognition as the major deficit in the high CU traits clusters is consistent with substantial research in clinic-referred disruptive as well as community samples of youth with varying levels of CU traits [2, 13], and in line with the concept of fearlessness that characterizes youth with low threat sensitivity [24]. Yet, our results imply that proactive aggression may not additionally contribute to poor fear recognition. Notably, it has been suggested that not the ability to recognize others’ facial expressions, but rather the ability to feel or empathize with someone else may be compromised in individuals showing high proactive aggression [15, 41].

Importantly, the cluster with high CU traits and reactive aggression but lower proactive aggression (‘CU-Reactive’) exhibited multiple difficulties in emotion recognition generally associated with reactive aggression and higher emotional reactivity. In particular, the increased tendency to interpret other emotions as anger in this cluster may point to a hostile attribution bias, which has been linked to reactive aggression following peer rejection [42]. Impaired sadness and disgust recognition has also been observed in offenders showing reactive violence reactive versus offenders showing proactive violence [14]. Notably, findings on disgust recognition have implicated the insula [43], with smaller volumes found to be associated with reactive but not proactive aggression as previously reported in this study sample [9]. Importantly, in contrast to the genetic correlation between CU traits and fear recognition, findings regarding sadness recognition suggested a role for environmental and individual exposures rather than genetic make-up [13], with other studies pointing to distinct environmental exposures for reactive versus proactive aggression [44]. These results suggest these exposures may differentiate between two our high CU traits clusters.

Our results suggest that working memory is involved in overall emotion recognition ability, which is in line with similar findings in disruptive behavior disorders [18] and across children with and without ADHD [45]. Although these results may in part be a result of task-specific demands (e.g., choosing the correct emotion out of six labels) [19], real world facial emotion recognition is a highly complex process demanding cognitive resources. Brain regions commonly involved in non-emotional cognitive functioning, including working memory, are active during emotion perception, pointing to the importance of working memory for handling information related to effectively processing social cues [46]. Notably, there is some evidence for the effectiveness of (emotional) working memory training in improving emotion recognition in borderline personality disorder, which is also characterized by emotionally impulsive aggression [47].

Working memory deficits in both high CU traits clusters do not support suggestions that higher executive functioning (of which working memory is an important aspect) may either protect against or facilitate the goal-directed nature of proactive aggression [21, 48]. Still, a large body of evidence supports the importance of poor working memory in the explanation of aggressive and antisocial behaviors through its role in self-regulation [49]. Possibly, CU traits, working memory and/or higher order cognitive functions and emotion recognition interact in complex ways with each other in their link to reactive and proactive aggression.

A strength of this study was our relatively large sample size due to our multi-site design. Moreover, we included a rather sophisticated statistical method to define homogeneous clusters of individuals based on CU traits and reactive and proactive aggression, using a sample of both clinic-referred and control youth, reflecting the fact that these traits exist on a continuum across the normal population and clinical samples. However, we did not include measures of the impulsivity-lifestyle and grandiose-deceitful dimensions of psychopathy beyond CU traits, which have been related to worse versus better emotion recognition [50], with the latter also often associated with proactive aggression [51]. In addition, our findings may not generalize to more severely affected samples, as even within clinic-referred youth the mean level of CU traits was relatively low in our sample compared to some previous studies. Yet, these studies were often pre-selected for high CU traits, thus not fully taking the dimensional nature of CU traits into account. Another limitation was the inclusion of relatively few females, not allowing to address sex-specificity, yet being characteristic of samples with disruptive behavior problems. Furthermore, the final number of individuals in some of the clusters used for emotion recognition and working memory analyses was relatively low. Still, phenotypic measures were compared between the full-sized clusters of moderate size, and remained similar when those missing the CANTAB measures were excluded. Finally, neutral or ambiguous expressions may be more appropriate for investigating the presence of a hostile attribution bias, although we were able to demonstrate an increased tendency to mistake other emotions for anger.

In conclusion, our results indicate that the presence of high CU traits and impaired fear recognition does not necessarily imply high proactive aggression. Instead, party distinct neurocognitive mechanisms in those with high CU traits and reactive aggressions and lower versus higher proactive aggression appear to exist, as indicated by more global emotion recognition deficits versus a specific deficit in fear recognition, respectively. Our study confirms CU traits as an important dimension along which youth with disruptive behavior may be characterized, but also stresses the importance of additionally considering reactive and proactive aggression. Our findings provide clues for improving prevention and intervention strategies, perhaps through emotion recognition training, which may especially benefit children with high CU traits and mild levels of proactive aggression [52]. Given the severity of proactive aggression, further research is warranted to identify neurocognitive risk and protective factors in youth with disruptive behaviors along various dimensions of psychopathic traits and aggression.

### Supplementary Information

Below is the link to the electronic supplementary material.

Supplementary file1 (DOCX 30 KB)
