# Supplementary material for: Emotion recognition profiles in clusters of youth based on levels of callous-unemotional traits and reactive and proactive aggression
Source: Eur Child Adolesc Psychiatry. 2022 Sep 20;32(12):2415–25. doi: 10.1007/s00787-022-02079-3 (PMC10682164; doi:10.1007/s00787-022-02079-3)
Supplement: Supplementary file 1 — Supplementary file1 (DOCX 30 KB) [file 787_2022_2079_MOESM1_ESM.docx]

| Characteristic | Low [1]  *n* = 54 | Low-Moderate [2]  *n* = 81 | CU-Reactive [3]  *n* = 76 | CU-Mixed [4]  *n* = 32 | Pairwise comparisons |
| --- | --- | --- | --- | --- | --- |
| *n* cases (%)^a^ | 11 (20%) | 39 (48%) | 69 (91%) | 30 (94%) | 3,4 > 2 > 1 |
| *n* male (%)^a^ | 30 (56%) | 55 (68%) | 66 (87%) | 27 (84%) | 3,4 > 1; 3 > 2 |
| *n* ODD (%)^a^ | 5 (9%) | 24 (30%) | 48 (63%) | 22 (69%) | 3,4 > 2 > 1 |
| *n* CD (%)^a^ | 3 (6%) | 7 (9%) | 48 (34%) | 15 (47%) | 3,4 > 1,2 |
| *n* ADHD (%)^a^ | 1 (2%) | 11 (14%) | 27 (36%) | 6 (19%) | 3,4 > 1; 3 > 2 |
| *n* medication (%)^a^ | 4 (8%) | 22 (27%) | 42 (56%) | 17 (53%) | 3,4 > 2 > 1 |
| age, *M (SD)^b^* | 13.2 (2.4) | 12.8 (2.6) | 12.8 (2.9) | 14.3 (2.5) | 4 > 2,3 |
| IQ, *M (SD)^b^* | 109 (12) | 103 (12) | 100 (11) | 98 (9) | 1 > 2 > 4 ; 1 > 3 |
| Aggression, *M (SD)^b^* | 54 (8) | 63 (13) | 73.5 (12) | 75 (11) | 3,4 > 2 > 1 |
| Rule-breaking, *M (SD)^b^* | 54 (6) | 59 (19) | 66 (10) | 69 (15) | 3,4 > 2 > 1 |
| ADHD symptoms, *M (SD)^b^* | 0.8 (2.5) | 3.0 (4.7) | 7.2 (5.8) | 4.4 (5.9) | 3 > 2,4 > 1 |
|  |  |  |  |  |  |
| **Clustering measures** |  |  |  |  |  |
| CU traits, *M (SD)^b^* | 14.5 (4.1) | 23.4 (5.7) | 30.4 (8.2) | 32.3 (8.0) | 3,4 > 2 > 1 |
| Reactive aggression, *M (SD)^b^* | 4.4 (2.3) | 8.2 (4.7) | 12.8 (3.4) | 14.9 (4.5) | 4 > 3 > 2 > 1 |
| Proactive aggression, *M (SD)^b^* | 0.0 (0.2) | 0.9 (0.8) | 4.2 (1.8) | 11.5 (4.2) | 4 > 3 > 2 > 1 |

**Supplementary Table 1.** Cluster characteristics total sample

ODD, Oppositional Defiant Disorder; CD, Conduct Disorder; CU, Callous-unemotional; ADHD, Attention Deficit/Hyperactivity disorder. Aggression and Rule-breaking as assessed by respective subscales of the Child Behavior Checklist (CBCL) [27]; CU traits as assessed by the Inventory of Callous-Unemotional traits (ICU) [29] Reactive and Proactive Aggression as assessed by the Reactive-Proactive Aggression Questionnaire RPQ) [4]. The clusters are numbered to indicate significant pairwise comparisons.
^a^ Cluster differences assessed by Pearson’s Chi Square test
^b^ Cluster differences assessed by one-way analyses of variance (ANOVA)
All pairwise comparisons significant at *p* < 0.05.
